# Comparative Studies of the Dynamics Effects of BAY60-2770 and BAY58-2667 Binding with Human and Bacterial H-NOX Domains

**DOI:** 10.3390/molecules23092141

**Published:** 2018-08-25

**Authors:** Rana Rehan Khalid, Muhammad Tahir ul Qamar, Arooma Maryam, Ayesha Ashique, Farooq Anwar, Mohammed H. Geesi, Abdul Rauf Siddiqi

**Affiliations:** 1 Department of Biosciences, COMSATS University Islamabad (CUI), Islamabad 45550, Pakistan; ray.binm@gmail.com (R.R.K.); aroomabinm@yahoo.com (A.M.); ayeshaashiq28@yahoo.com (A.A.); 2 College of Informatics, Huazhong Agricultural University (HZAU), Wuhan 430070, China; m.tahirulqamar@webmail.hzau.edu.cn; 3 Department of Chemistry, University of Sargodha, Sargodha 40100, Pakistan; fqanwar@yahoo.com; 4 Department of Chemistry, College of Sciences and Humanities, Prince Sattam Bin Abdulaziz University, Al Kharj 11942, Saudi Arabia

**Keywords:** soluble guanylate cyclase, sGC activator compounds, quantum calculation, molecular dynamics simulation

## Abstract

Soluble guanylate cyclase (sGC) is a key enzyme implicated in various physiological processes such as vasodilation, thrombosis and platelet aggregation. The enzyme’s Heme-Nitric oxide/Oxygen (H-NOX) binding domain is the only sensor of nitric oxide (NO) in humans, which on binding with NO activates sGC to produce the second messenger cGMP. H-NOX is thus a hot target for drug design programs. BAY60-2770 and BAY58-2667 are two widely studied activators of sGC. Here we present comparative molecular dynamics studies to understand the molecular details characterizing the binding of BAY60-2770 and BAY58-2667 with the human H-NOX (*h*H-NOX) and bacterial H-NOX (*b*H-NOX) domains. HartreeFock method was used for parametrization of both the activators. A 50 ns molecular dynamics (MD) simulation was run to identify the functionally critical regions of the H-NOX domains. The CPPTRAJ module was used for analysis. BAY60-2770 on binding with *b*H-NOX, triggered rotational movement in signaling helix F and significant dynamicity in loops α and β, but in *h*H-NOX domain the compound showed relatively lesser aforementioned structural fluctuations. Conversely, *h*H-NOX ligated BAY58-2667 experienced highest transitions in its helix F due to electrostatic interactions with D84, T85 and R88 residues which are not conserved in *b*H-NOX. These conformational transformations might be essential to communicate with downstream PAS, CC and cyclase domains of sGC. Comparative MD studies revealed that BAY bound *b*HNOX dynamics varied from that of *h*H-NOX, plausibly due to some key residues such as R40, F74 and Y112 which are not conserved in bacteria. These findings will help to the design of novel drug leads to cure diseases associated to human sGC.

## 1. Introduction

Soluble guanylate cyclase is a heterodimeric enzyme of 150 kDa molecular mass which consists of two subunits—alpha (α) and beta (β) [1]. Of several isoforms of α and β subunits, the α1β1 and α2β1 are predominantly expressed heterodimers of sGC in mammalian cells [2]. Starting from N-terminus, the β subunit folds into H-NOX, PAS, coiled coil (CC) and catalytic domains; the α subunit also follows the same architecture, however its N-terminal does not bind with heme and istherefore termed as pseudo-H-NOX domain [3]. The β H-NOX harbors a histidine bound heme molecule which is capable of bindingnitric oxide (NO) with femtomolar sensitivity and has been also been found to bind with other gaseous ligands such as O_2_ and CO, though with much lesser sensitivity than that with NO [4,5,6,7]. The sensitivity of sGC is high when its heme moiety is reduced i.e., when the heme iron is in Fe^2+^ state. High concentration of cellular reactive oxygen species oxidize the heme (Fe^3+^) disabling it from capturing small gaseous ligands which in turn leads to sGC inactivation [2]. sGC is known as the only receptor of NO in mammals, which regulates many physiological responses such as vasodilation, smooth muscle relaxation, thrombosis, platelet aggregation and inhibition of inflammation [8,9]. In mammals, cellular NO is produced by nitric oxide synthases (NOS). On NO binding, H-NOX gets activated which in-turn communicates with PAS and CC domains to activate the cyclase domain for the conversion of a GTP molecule into cGMP. The cGMP serves as second messenger playing significant role in the regulation of many downstream. sGC is therefore a hot target for designing novel drugs to cure the disorders associated to cGMP signaling pathway such as peripheral hypertension, pulmonary arterial hypertension (PAH), heart failure and liver fibrosis [10,11]. 

**Figure 1:**
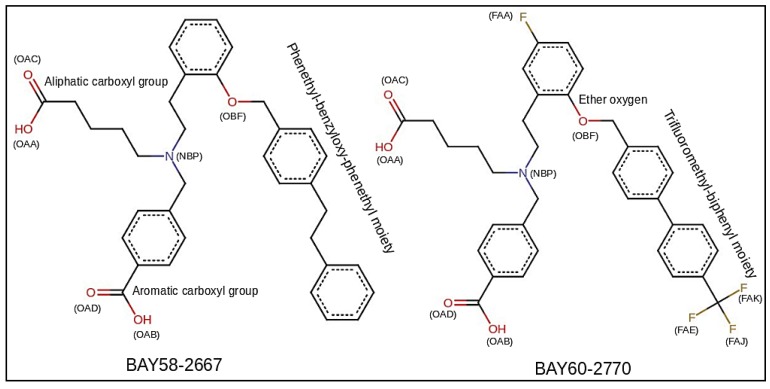
BAY activators, BAY58-2667 (4-[[(4-carboxybutyl)[2-[2-[[4-(2-phenylethyl)phenyl]methoxy] phenyl] ethyl]amino]methyl]benzoic acid) and BAY60-2770(4-({(4-carboxybutyl)[2-(5-fluoro-2-{[4’-(trifluoromethyl)biphenyl-4-yl]methoxy}phenyl)ethyl]amino}methyl)benzoic acid) used in this study; conventional names and chemical structure of both the activators are also given. Aliphatic and aromatic carboxyl groups are illustrated as OAC, OAA, and OAD, OAB respectively; Fluorine atoms of BAY60-2770’s fluoro and trifluoromethyl-biphenyl moieties have been represented as FAA, FAE, FAJ, and FAK.

Experimental and clinical studies have shown that impaired bioavailability of NO contributes to cardiovascular, endothelial, hepatic and pulmonary dysfunctions [12]. Organic nitrates (sodium nitroprusside, nitroglycerin) have been used as nitrovasodilators for the treatment of cardiovascular disorders, but their utilization has some limitations such as severe hemodynamic effects including reduced bioavailability, lack of selectivity, tolerance and insufficient metabolism [13]. Therefore, therapeutic approaches sought for discovering novel modulators which could trigger sGC to enhance cGMP production. The sGC modulators have two classes, stimulators and activators. The sGC stimulators stimulate sGC directly i.e., when its heme iron is reduced (Fe^2+^) but NO synthesis is impaired or enzyme’s NO sensitivity is compromised. These include YC-1, BAY41-8543, BAY63-2521 (riociguat) and BAY41-2272 [2,14,15]. Among sGC stimulators, riociguat has already been approved by FDA for the treatment of pulmonary hypertension [16,17,18]. sGC activators, such as HMR-1766, BAY58-2667 and BAY60-2770, bind to sGC and activate it in a NO- and heme-independent manner i.e., when heme iron of sGC has been oxidized (Fe^3+^) or heme has been lost by the enzyme [2,19]. Recently sGC activators have drawn attention of researchers as during oxidative stress, when the enzyme becomes inactive i.e., its heme iron is oxidized by ROS or RNS (Reactive Nitrogen Species), stimulators like riociguat cannot elicit the cyclase activity [20,21]. Among the activators, BAY58-2667 (cinaciguat) is already in clinical development by Bayer AG and has been found to elicit vasodilation while preserving the kidney function, although it has been reported to cause hypotension [22,23,24]. The drug has been also found to result a long-lasting antihypertensive effect and inhibit platelet aggregation and ischemia [25,26]. The therapeutic potential of BAY60-2770 has been also demonstrated against hypertension in rat, erectile dysfunction in obese mice, platelet aggregation in humans, and asthma in mice [27,28,29,30,31,32]. Both BAY58-2667 and BAY60-2770 mimic the heme moiety, (Figure 1). Both BAY activators have two charged hydrophilic carboxylate groups which are crucial to interact with the Y-S-R motif of H-NOX like heme carboxylate group. The only difference between the two activators is their hydrophobic tail. These trifurcated activators behave somewhat like heme moiety. Cinaciguat’s tri-benzyl tail establishes hydrophobic binding modes with L4, W74, T78, K83, F97, L101, L104, V108, L148, and L152 residues in bacteria. In contrast to BAY58-2667, the hydrophobic tail of BAY60-2770 extends with fluoro and trifluoromethyl-biphenyl moieties which contribute to extensive interactions with binding pocket residues such as Y2, Y83, S111 and F112 [11,22].

In this study, we seek to reveal the dynamics effects of sGC activators BAY58-2667 and BAY60-2770 on bacterial and human H-NOX domains (*b*H-NOX and *h*H-NOX). The study aims to enhance our understanding of binding pocket behavior against the activator compounds in both the biological systems (bacteria and humans) and observe the most prominent dynamic events that might be involved in signal transduction for cGMP production. Stability of activator bound *b*H-NOX and *h*H-NOX complexes was studied by 50 ns MD simulations. We have observed that there are some non-conserved residue positions in the binding pocket that may affect the dynamics of H-NOX in both systems. This study would therefore provide further insight for designing drug screening projects against H-NOX.

## 2. Results and Discussion

### 2.1. Comparative Modeling

**Figure 2:**
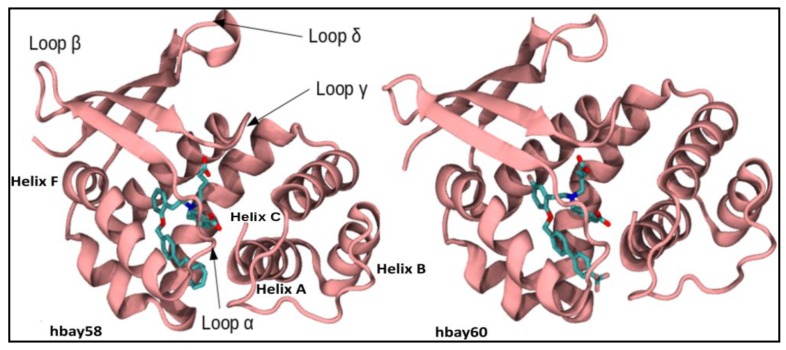
Models of *h*H-NOX bound with BAY58-2667 (hbay58) and BAY60-2770 (hbay60). *h*H-NOX domain consists of seven α helices and four β strands. Important secondary structure elements have been shown.

Crystal structures of Nostoc H-NOX domains bound to BAY60-2770 (PDB ID: 4IAE) [11] and cinaciguat (BAY58-2667) (PDB ID: 3L6J) [22] were used as a template to model the *h*H-NOX domain. *h*H-NOX is encoded by first 186 N-terminal residues of the β subunit of sGC. Query sequence showed 34 percent identity with the template. Statistical parameters such as z-DOPE score [33], GA341 [34], TM-score [35] were employed to evaluate and validate the predicted *h*H-NOX models. All the three metrics were found to lie in the most favorable ranges: z-DOPE was found to be −1.04 [36], fold reliability GA341 was 1 (100% reliable predicted fold) and global structure quality TM-score was found to be 0.83 (Figure 2). A Ramachandran plot of the initially predicted model showed that 94.6% residues were in the favored region while 97.3% residues lied in the allowed region [37]. These models were then subjected to refinement through YASARA [38]. After the refinement, 98.3% residues of the models were observed to lie in the favored region and 99.4% in the allowed region. 

**Figure 3:**
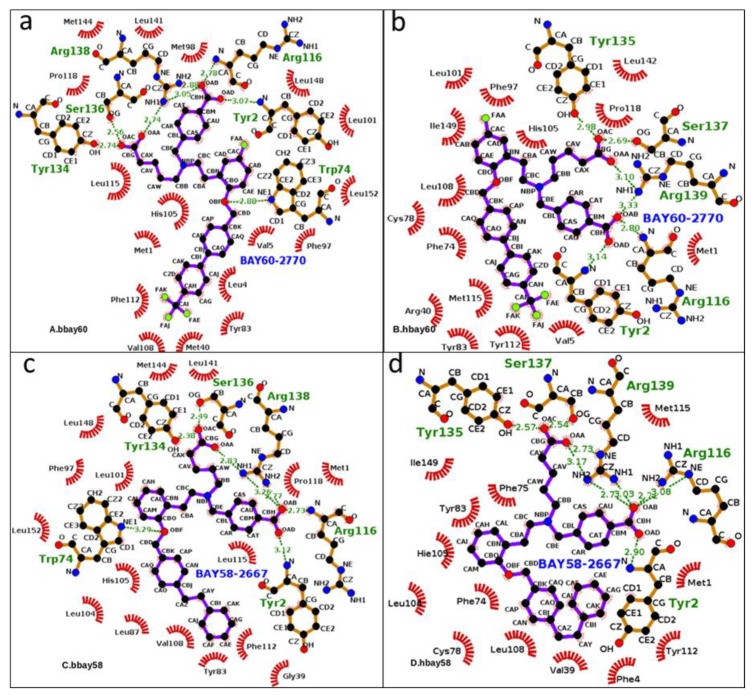
From **a** to **d**, 2D graphical outlay of binding modes of sGC activators bound in the binding pockets of human and bacterial H-NOX domains (bbay60, hbay60, bbay58, hbay58). The energy minimized starting structures (hbay58 and hbay60) along with bbay58 and bbay60 (crystal structures) were subjected to MD production phase. Green dotted lines represent Hydrogen bonds whereas hydrophobic interactions are shown by red archs, the ligand atoms are shown in blue and black ball and stick model whereas protein atoms are shown in brown (stick) and black (ball). Aliphatic and aromatic carboxyl groups atoms of activators (OAA, AAB, OAC, OAD) exhibited prominent hydrogen bond interaction with functionally critical residues such as Y2, R116, Y135, S137, R139 in *h*H-NOX andY2, W74, R116, Y134, S136, R138 in *b*H-NOX.

The complexes, BAY58-2667 bound *b*H-NOX and *h*H-NOX (bbay58 and hbay58, respectively) and BAY60-2770 bound *b*H-NOX and *h*H-NOX complexes (bbay60 and hbay60, respectively), were submitted to Ligplot tool [39], to visualize the binding modes of initial models. Figure 3 describes the interaction network of the energy-minimized BAY58-2667 and BAY58-2667 bound structures of *h*H-NOX and *b*H-NOX (hbay60 and hbay58). 

### 2.2. Molecular Dynamics Simulation

**Figure 4:**
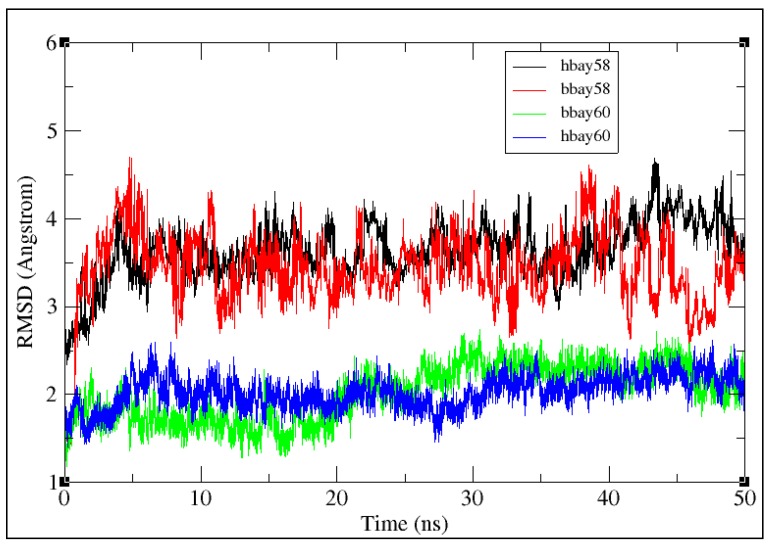
50 ns RMSD trajactories of the backbone atoms of BAY58-2667 and BAY60-2770 bound *b*H-NOX and *h*H-NOX complexes (bbay58, hbay58, bbay60 and hbay60). Y-axis represents the value of RMSD in Angstroms (Å) and the X-axis shows the duration of the simulation trajectory in nanoseconds (ns). All the four trajectories are traced in different colors, BAY58-2667 bound *h*H-NOX (hbay58) is represented in black; BAY58-2667 bound *b*H-NOX (bbay58) is shown in red; BAY60-2770 bound *b*H-NOX (bbay60) is illustrated in green and BAY60-2770 bound *h*H-NOX (hbay60) is shown in blue.

To evaluate the conformational stability of BAY58-2667 bound *b*H-NOX and *h*H-NOX (bbay58 and hbay58, respectively) and BAY60-2770-bound *b*H-NOX and *h*H-NOX complexes (bbay60 and hbay60, respectively), root mean square deviation (RMSD), root mean square fluctuation (RMSF) were investigated for all backbone atoms whereas the radius of gyration (RoG) was examined for all-atoms through CPPTRAJ [40]. Figure 4 shows 50 ns RMSD trajectories of bbay58, hbay58, bbay60 and hbay60. Both bbay58 and hbay58 showed higher RMSD values across entire trajectory of 50 ns than bbay60 and hbay60 with an average RMSD of 3.8 Å. RMSD values of bbay58 and hbay58 structures also varied significantly during the last 10 ns of simulation, showing instable binding behavior of BAY58-2667 with *b*H-NOX. Whereas trajectories of bbay60 and hbay60 showed much lower RMSD with an average deviation of 2 Å across the entire 50 ns trajectory. In contrast to those of bbay58 and hbay58, both the bbay60 and hbay60 trajectories followed the similar behavior exhibiting the stability of BAY60-2770 bound structures. An evident deviation in RMSD behavior was noticed which may be attributed to ligand type and not to the biological system (*b*H-NOX, *h*H-NOX). These results are consistent with earlier findings which suggested that trifluoromethyl-biphenyl hydrophobic tail of BAY60-2770 was responsible for increased stability and fold integrity of BAY60-2770 bound complex of Nostoc H-NOX [11]. 

**Figure 5:**
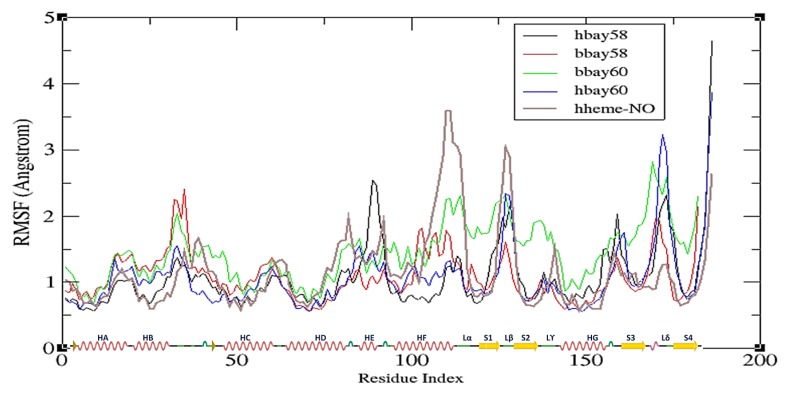
Average fluctuation experienced by each H-NOX residue, backbone atoms, represented as root mean square fluctuation or RMSF traces: hbay58 (black), bbay58 (red), bbay60 (green), hbay60 (blue) and NO bound heme Fe^2+^-*h*H-NOX or hheme-NO (purple) over the course of 50 ns simulation. Y-axis shows RMSF in Angstroms Å and X-axis represents H-NOX residues from N to C terminus (residue index). The X-axis also shows secondary structure layout of the H-NOX structure, helices are represented as red springs, loops as green lines, turns as green curves and strands as yellow arrows.

Figure 5 shows the RMSF plots for the four ligand bound complexes delineating the net 3D movement of all residues of *b*H-NOX and *h*H-NOX of binding with BAY58-2667 and BAY60-2770. RMSF trajectories of the four complexes have also been compared with that of *h*H-NOX bound with NO (hhme-NO). The RMSF plot describes that in hbay58 the residues 88–91 (HE), 125–129 (loop β) and 171–174 (loop δ) show fluctuations at 2.6 Å, 2 Å and 2.4 Å, respectively. These fluctuations play a significant role in the activation of sGC enzyme; previous studies also demonstrated that on NO binding loops β and δ of H-NOX show substantial displacements [41]. In bbay58 fluctuations of 2.4 Å, 1.3 Å, and 1.8 Å across loop segment between helices B and C (a.a. 31–35), helix F (a.a. 103–112) and loop δ (a.a. 171–175) were observed respectively. The bbay58 complex behaved slightly different as compared to hbay58 because some BAY58-2667 binding residues in the binding pockets of *b*H-NOX and *h*H-NOX are not conserved. Supposedly, the RMSF peaks observed across helix F owe to its earlier observed rotational movement [41], which displaces the following loop regions away from their original position; helping to activate sGC [41]. In hbay60, the region spanning through helix F to loop β and loop δ showed on average 2.4 Å and 3.4 Å fluctuations respectively. Loop δ fluctuation was significantly high among all systems and the fluctuation pattern was observed to be quite similar to that exhibited by NO bound *h*H-NOX complex (hheme-NO). Thus it might be possible that loop δ residues transduce signals to neighboring PAS and CC domains for the onward communication to sGC cyclase domain. In case of hbay60, BAY60-2770 binding elicited higher fluctuations in the loop β and δ residues than those observed in hbay58. Conversely, bbay60 showed fluctuations across loop segment between helices B and C (a.a. 31–35), loop α (a.a. 110–116), β (a.a.125–130) and δ (a.a. 170–174) measuring 2 Å, 2 Å, 2 Å and 2.8 Å, respectively. In both bbay60 and hbay60, loop δ showed considerably high fluctuations.

**Figure 6:**
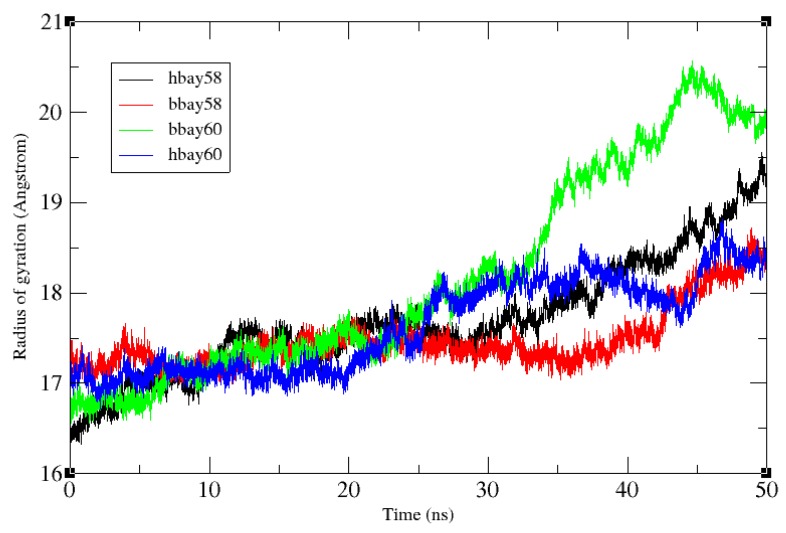
All-atoms radius of gyration (RoG) variations over 50 ns time period in case of hbay60, bbay60 and hbay58 and bbay58, all the four systems remained compact, with a consistent RoG of 17.5 Å for the first half of the simulation i.e., 25 ns. RoG was observed to loose consistence post 25 ns phase, bbay60 showing the highest rise of RoG ending at 20.5 Å. followed by hbay58 (19 Å), hbay60 (18.5 Å) and bbay60 (18.5 Å).

All the systems remained compact up to 25 ns with the average radius of gyration (RoG) of 17.3 Å (Figure 6). After 25 ns, bbay60 lost compactness rising to as much as 20.5 Å by the end of simulation, showing highest fold movement amongst all the systems. Subsequently, the binding pocket slightly opened causing the water molecules to infiltrate in the protein’s hydrophobic core, thereby increasing the surface accessibility and ligand interactions. Average RoG for Bay60-2770 bound *h*H-NOX (hbay60) remained 17.5 Å for first 25 ns rising to 18 Å in the end, signifying moderate fold movement in the event of Bay60-2770 binding with *h*H-NOX. In case of hbay58, the RoG rose gradually to 19.5 Å, after remaining consistent at 17.5 Å for the first 25 ns. The complex bbay58 was found to be the most compact structure amongst all the systems showing consistent 17.5 Å RoG for the first 42 ns ending at RoG of 18 Å implying that the *b*H-NOX fold remained intact most of the time with a little movement in the end. High RoG in case of bbay60 contributes to the outward movement of the *b*H-NOX’s proximal side of the binding site and higher fluctuation of loop region between helices B and C. Rise of RoG during 26–50 ns was due to large movements by loop δ as observed in RMSF analysis. 

### 2.3. Hydrogen Bond Analysis

**Figure 7:**
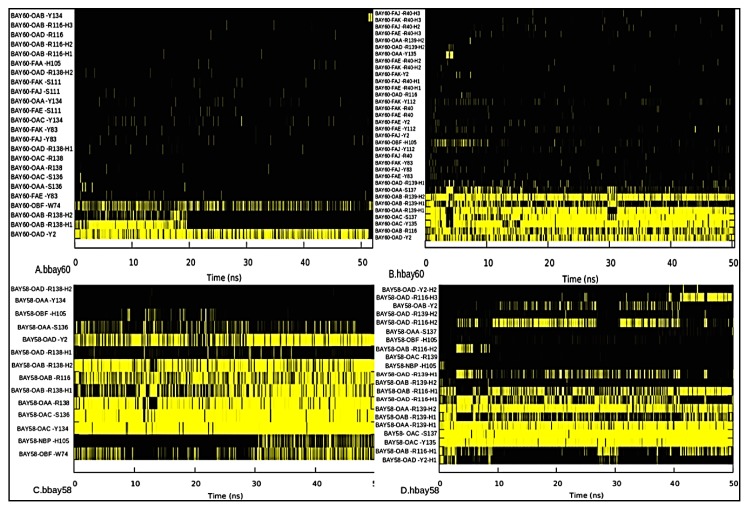
Hydrogen bond occupancy as observed in bbay60 (**A**), hbay60 (**B**), bbay58 (**C**) and hbay58 (**D**) complexes during 50 ns simulation period, outlaid at X-axis. Y-axis shows hydrogen bond forming atoms of the BAY compounds and binding residues of H-NOX, the atom abbreviations are same as illustrated in Figure 1. Hydrogen bonds were recorded throughout the 50 ns trajectory in all the four complexes. Yellow bars indicate the event of hydrogen bond formation as observed at that point of time at X-axis. Length of the yellow trace illustrates the occupancy or the time duration for which the respective hydrogen bond was observed.

Hydrogen bond analysis was performed to investigate stability and persistence (occupancy) of hydrogen bonds between BAY compounds and key residues of the binding pockets of *b*H-NOX and *h*H-NOX over the 50 ns simulation [42]. Gnuplots at panels A, B, C and D (Figure 7) for bbay60, hbay60, bbay58 and hbay58 respectively show that carboxylate group atoms, OAA, AAB, OAC and OAD, of both the activators behave like heme propionate moiety forming strong and stable hydrogen bonds with Y2, R116, and Y-S-R (Y135-S137-R139) motif of *b*H-NOX and *h*H-NOX. This interaction was observed in both of the biological systems, but BAY58-2667 hydrogen bond fraction values are high as compared to those observed with BAY60-2770. Figure 7A reveals that BAY60-2770 fluoro and trifluoromethyl-biphenyl moiety atoms, FAA, FAE, FAJ and FAK, exhibit few hydrogen bond interactions with binding pocket residues Y83 and S111 in case of bbay58; however in hbay60, these atoms show electrostatic attractions for Y2, R40, Y83 and Y112 residues (Figure 7B). Generally, more stable hydrogen bonding has been observed in hbay60. These results indicate that trifluoromethyl-biphenyl moiety of BAY60-2770 favors the formation of a stable complex with binding site residues of *h*H-NOX than those of *b*H-NOX. In bbay58 complex, ether oxygen (OBF) shows strong hydrogen bond interaction with W74. The substitution of bacterial W74 with F74 in *h*H-NOX contributes to slight displacement of the bound BAY compounds towards proximal side of the binding pocket. Therefore, the hydrogen bond between ether oxygen (OBF) and H105 might likely restrict the signaling helix F specifically in hbay60. Further investigation showed that in hbay60, Y2 showed strong hydrogen bond interaction with trifluoromethyl-biphenyl moiety atoms which was absent in bbay60 (Figure 7A,B). The hydrogen analysis also explained an earlier finding that F112 mediates the rotational movement of helix F in *b*H-NOX [15]. The *b*H-NOX F112 shows strong proximal site interactions with surrounding residues such as V108, L115, G109, Q114, and S111 (Table 1). Generally the hydrogen bond forming atoms were the same in hbay58 and bbay58 and bbay60 and hbay60 with the exception of those involved in bonding with trifluoromethyl-biphenyl moiety (Figure 7). Therefore, the aforementioned analysis implies that tail of BAY58-2776 shows more flexibility as compared to that of BAY60-2770. 

Key residues involved in hydrogen bond network across binding sites of bbay60, hbay60, bbay58 and hbay58 were also studied. We observed that residues V108, F112, G109, S111 and L115 in case of *b*H-NOX, and Y2, Y83, L108, Y112 and M112 in case of *h*H-NOX were involved in hydrogen binding network across binding sites. However, position 112 was specifically crucial as residues occupying this position both in *b*H-NOX and *h*H-NOX, F112 and Y112 respectively, were involved in most of the binding pocket hydrogen bonds over the entire path of the 50 ns trajectory (Table 1). Thus, the aforesaid residues were found to be the key determinants for the binding pocket integrity and complexation with the BAY compounds allosterically.

### 2.4. Principle Component Analysis

**Figure 8:**
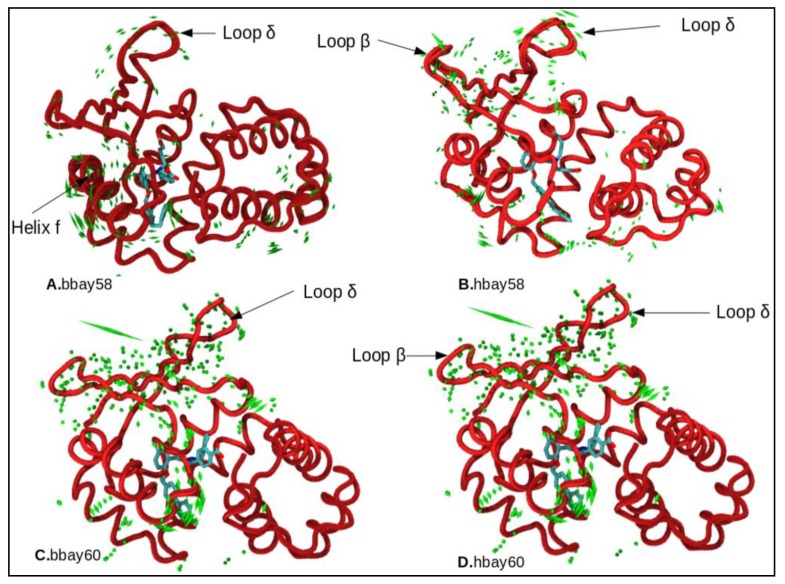
Uncorrelated trajectories Analysis through essential dynamics depicted by the porcupine plot of (**A**) bacterial H-NOX bound BAY58-2667 (bbay58); (**B**) human H-NOX bound BAY58-2667 (hbay58); (**C**) bacterial H-NOX bound BAY60-2770 (bbay60); and (**D**) human H-NOX bound BAY60-2770 (hbay60) respectively. Green color porcupines represent those regions which experienceconsiderable movement. All dynamically important regions which showed changes during principle component analysis are further highlighted with arrows.

Principle component analysis (PCA) was undertaken to investigate the dynamics of BAY58-2667 and BAY60-2770 binding with *b*H-NOX and *h*H-NOX domains. PCA results indicated that hbay58 showed highest movement across loop β (a.a. 124–129), and relatively lesser movement across loop δ (a.a.169–177) region (Figure 8B). Uncoiling of small β fragment across residues 83–91 was also observed due to electrostatic interactions between BAY atoms and the *h*H-NOX residues R40, D84, T85 and R88, not found in *b*H-NOX (Figure 8A,B). The activation signal may thus be transduced through loop β and the loop δ as these regions showed significant movements. Previous dynamics studies of post NO binding activation mechanism of sGC described the significance of helix F, and loop β [41], but the dynamic changes in drug bound systems were not studied. Here we found that helix F did not show massive displacement thus the binding cavity did not open as much in BAY bound H-NOX as in NO bound H-NOX (Figure 8B,D). In bbay58, helix F showed a slight rotational movement, and this movement was suggestively driven by the presence of F 112. That much rotational movement of helix F was not observed in hbay58 and hbay60, putatively due to residue Y112 in *h*H-NOX which showed strong interactions with Y2, R40, and Y83 residues, thus limiting the rotational movement of helix F in *h*H-NOX. Therefore, in case of hbay60 minimal movement was observed in loops α, β, γ, δ and helix F. 

Our analysis reveals that though BAY compounds cannot activate *h*H-NOX as efficiently as in case of NO-bound *h*H-NOX, these compounds can generate the required dynamics capable to activate sGC considerably. This is particularly important in case of oxidized (inactive) state of H-NOX [43,44]. The findings of this study also suggest that dynamicity in loop β in *h*H-NOX is the key signal which leads to the cascade of dynamic events through loop δ and helix-F, ultimately culminating into an activated *h*H-NOX. As these secondary structure elements lie near the c-terminal or proximal end of the H-NOX domain, putatively the aforesaid dynamic events also serve to communicate the downstream activation signal through interacting with the neighboring PAS domain of sGC. A relatively lesser movement of helix F has been observed in case of hbay58 and hbay60 compared to that observed in corresponding bacterial BAY bound complexes. This is an interesting finding, earlier in-vitrostudies [7] have demonstrated that *h*H-NOX is the most sensitive NO sensor in the entire H-NOX family. Therefore even a lesser dynamicity characterized by the BAY binding with *h*H-NOX, than that observed in case of NO binding, might be enough to elicit the activation reported by earlier studies carried out in rats, mice and humans [22,23,24,25,26,27,28,29,30,31,32]. These findings highlight the importance of targeting other regions of H-NOX to explore potential allosteric sites which may help designing drugs for NO-independent activation of sGC. This study also elucidates in detail the mechanism of the BAY activators’ binding with *b*H-NOX and *h*H-NOX, comparing the key residues involved in the architecture of heme binding sites and their varied roles in H-NOX activation.

## 3. Materials and Methods

### 3.1. Comparative Modeling

sGC activators (BAY58-2667, BAY60-2770) bound *b*H-NOX domains PDB ID: 3L6J [22] and PDB ID: 4IAE [11] were used as a reference for comparative dynamics studies with *h*H-NOX domain. Human beta H-NOX target sequence showed 34% sequence identity, 51% sequence homology and 99% query coverage with Nostoc H-NOX domain with a bit score of 105, which was thus chosen as a template structure for comparative modeling; PDB structures 3L6J and 4IAE were used for predicting *h*H-NOX domains. Modeller 9.16 [45] was employed for homology modeling, best models were selected on the basis of z-DOPE score, RMSD, and global structure quality. The predicted models were validated through RAMPAGE [46]. Energy minimizations and refinements of initial models were done through YASARA [38]. MolProbity server was used to validate all the stereochemical characteristics of predicted models [37]. Pymol builder module was used to add an NO molecule bonded with Fe^2+^ of heme prosthetic group of *h*H-NOX domain [47].

### 3.2. sGC Activators Charge Derivation

Restrained electrostatic potential charge calculation was done for BAY58-2667 and BAY60-2770 compounds, Antechamber17 [48] was used to produce Gaussian input file for quantum mechanics calculations. The HartreeFock method and 6-31G* basis set were used for optimization and charge calculation [49]. The first and second stage RESP was calculated by using Antechamber17 program, prep file for non-stranded residues was generated for parametrizing complete H-NOX drug bound systems.

### 3.3. Molecular Dynamics Simulation

sGC activator systems, which included *h*H-NOX bound with BAY58-2667, BAY60-2770 (hbay58, hbay60) and *b*H-NOX bound with BAY58-2667, BAY60-2770 (bbay58, bbay60) and NO bound *h*H-NOX, were subjected to molecular dynamics simulation. FF14SB force field was used for receptor protein in Amber14 [50]. The orthogonal box was used to immerse the systems with TIP3P water model [51]. Tleap was employed for adding missing hydrogen atoms and neutralizing the systems. All the five systems were enabled for periodic boundary conditions [52]. The cutoff was set 10 Å for long-range electrostatics interactions (PME) [53]. Parametrization of *h*H-NOX domain active site (NO-heme-H105) was accomplished through quantum calculation (Gaussian 09).B3LYP theory and 6-31G* basis set were implemented for the optimization and force constant calculation. After completing parameterization, all the systems were accounted for energy minimization (20,000 steps) to remove the steric clashes. Langevin thermostat was used for annealing the systems from 0 to 300 K at constant volume for 200 ps. Moreover, all four models were run for 50 ns during the production phase. SHAKE [54] was applied to constrained covalent bond containing hydrogen. All the simulations process was run with the integration step of 2 fs. We used CPPTRAJ v17.00 [40] for post-processing trajectories analysis which included fundamental stability analysis (RMSD, RMSF, and ROG) as well as more advanced analyses like hydrogen bond analysis and principal component analysis (PCA). VMD [55] was used for visualization of trajectories in real time, while Gnuplot [42] and Xmgrace [56] tools were used for graphical representations of plots.

### 3.4. Hydrogen Bond Occupancy

Hydrogen bonding has an essential role in the folding of proteins CPPTRAJ v17.00 was applied to estimate the solute-solute, solute-solvent and solute solvent-solute bridging hydrogen interactions of all human and bacterial H-NOX bound BAY compounds (hbay58, hbay60, bbay58, bbhay60) [57]. Hydrogen bond interactions of BAY60-2770 and BAY58-2776 with *b*H-NOX and *h*H-NOX binding pocket and distal and proximal residues were also analyzed. Hydrogen bonds among all the four systems were compared to explain different dynamic events attributed to hydrogen bond occupancy. Gnuplot and Xmgrace were used to analyze hydrogen bond occurrence and its lifetime (fraction).

### 3.5. Principal Component Analysis

Before undertaking principal component analysis, CPPTRAJ v17.00 module was employed to strip solvent and ions from 50 ns long MD trajectories. These modified striped trajectories were aligned over minimized reference structure. All backbone atoms were used as a mask to perform PCA [57]. PCs of the four systems were determined by diagonalizing the covariance matrix which generates eigenvalues. MD trajectories were then projected over these eigenmodes. We noticed that first three PCs cover entire motion of 50 ns long trajectories. Principle component analysis scatters plots were then generated using Xmgrace. VMD was used to visualize the uncorrelated systems movements more conclusively and to create all structural diagrams. ProDy interface of Normal mode wizard was applied to generate porcupine plot [58].

## 4. Conclusions

The carboxylate groups of both the BAY compounds maintained similar interaction patterns with the Y-S-R motif and R116 residue of H-NOX as exhibited by heme. Their ether oxygen shows a strong interaction with W74 in the *b*H-NOX system, but this residue is not conserved in *h*H-NOX due to which oxygen forms astrong interaction with H105. This interaction is shifted towards the proximal side of the binding site of *h*H-NOX. Both BAY compounds caused high fluctuation in the loop region between helix B and C in case of *b*H-NOX. It may be due to M40 which showed no interaction with its Y83, R88, and 112Y residues while in *h*H-NOX, R40 showed strong interactions with those residues. BAY58-2667 triggered small rotational movement in helix F which is vital for sGC activation. Conversely, when BAY60-2770 binds with *h*H-NOX, the helical fragment remains stable because of the interaction of trifluoromethyl-biphenyl moiety with Y2, R40, Y83 residues. It was found that BAY60-2770 generated considerable movement in loops β and δ; especially in *h*H-NOX, loop δ movement was highest among all systems. BAY58-2667 precisely rotates the helix F and elicits loop β fluctuations.Based on the simulation analysis of aforementioned complexes, we can propose that helix F, loops β and δ may be involved in signal transduction mechanism from H-NOX to adjacent PAS domains, but this dynamics pattern differsfrom NO bound H-NOX activation dynamics in which helix F, loop β movement was found to be several folds higher. Target-based drug discovery approaches can be used to modify the BAY compounds to make them more specific and selective to human sGC.

## Figures and Tables

**Table 1:** Key residues involved in the hydrogen bonding network across binding pockets of bbay60, hbay60, bbay58 and hbay58 complexes. Hydrogen bond forming atoms: acceptor, donor hydrogen and donor atoms are also listed. The occupancy of a bond-hydrogen is given as percent of MD frames reporting the bond. Average distance of each H bond is also given as measure of the bond strength.

Complexes	Acceptor	Donor H	Donor	Percent of Frames of 50 ns Simulation Showing the Hydrogen Bond	Average Distance Å
bbay60	V108@O	F112@H	F112@N	24.58	2.88
	F112@O	L115@H	L115@N	18.52	2.91
	G109@O	F112@H	F112@N	2.24	2.91
	S111@O	F112@H	F112@N	0.44	2.83
hbay60	L108@O	Y112@H	Y112@N	69.93	2.85
	Y112@O	M115@H	M115@N	15.27	2.91
	Y83@OH	Y112@HH	Y112@OH	8.02	2.82
	Y112@O	Y2@HH	Y2@OH	0.20	2.77
bbay58	V108@O	F112@H	F112@N	37.43	2.87
	F112@O	L115@H	L115@N	12.50	2.92
	G109@O	F112@H	F112@N	2.96	2.91
	F112@O	Q114@H	Q114@N	0.10	2.83
	F112@O	Y2@HH	Y2@OH	0.10	2.81
hbay58	L108@O	Y112@H	Y112@N	58.64	2.87
	Y112@OH	Y83@HH	Y83@OH	8.59	2.83
	Y112@O	M115@H	M115@N	6.80	2.92
	Y83@OH	Y112@H	Y112@OH	1.63	2.81
	V39@O	Y112@HH	Y112@OH	1.56	2.79
	Y112@OH	R40@HE	R40@NE	0.21	2.90
	Y112@OH	R40@HH12	R40@NH1	0.10	2.89
